# *Chlamydia Trachomatis* Infection-Associated Risk of Cervical Cancer: A Meta-Analysis

**DOI:** 10.1097/MD.0000000000003077

**Published:** 2016-04-01

**Authors:** Haiyan Zhu, Zhaojun Shen, Hui Luo, Wenwen Zhang, Xueqiong Zhu

**Affiliations:** From the Department of Obstetrics and Gynecology, the Second Affiliated Hospital of Wenzhou Medical University, Wenzhou, China.

## Abstract

As whether *Chlamydia trachomatis* infection increases the risk of cervical cancer is controversial in the literature, we performed a meta-analysis.

Based on a comprehensive search of publications in the Medline, Cochrane, and EMBASE databases, we identified and extracted data from all relevant articles examining *C. trachomatis* infection and the risk of cervical cancer. The quality of each included study was assessed according to the 9-star Newcastle–Ottawa scale. The strength of association between the *C. trachomatis* and risk of cervical cancer was estimated by odds ratio (OR) and 95% confidence intervals (CIs). This review was registered at PROSPERO with registration No. CRD42014015672.

A total of 22 studies with 4291 cervical cancer cases and 7628 controls were identified. Overall, *C. trachomatis* was significantly linked to increased cervical cancer risk in prospective studies (OR = 2.21, 95% CI: 1.88–2.61, *P* < 0.001), as well as in retrospective studies (OR = 2.19, 95% CI: 1.74–2.74, *P* < 0.001). Additionally, with a multivariate logistic regression analysis adjusted for HPV and age, *C. trachomatis* infection was identified as an independent predictor of cervical cancer in 11 studies (OR = 1.76, 95% CI: 1.03–3.01, *P* = 0.04). Coinfection of human papilloma virus and *C. trachomatis* has a higher risk of cervical cancer (OR = 4.03, 95% CI: 3.15–5.16, *P* < 0.001). A subgroup analysis based on histological type indicated an elevated risk for both squamous cell carcinoma (OR = 2.21, 95% CI: 2.00–2.45, *P* < 0.001), and adenocarcinoma (OR = 1.61, 95% CI: 1.21–2.15, *P* = 0.001), in associated with *C. trachomatis*. Subgroup analysis by where *C. trachomatis* infection was detected showed a significantly higher risk of cervical cancer associated with *C. trachomatis* infection detected in serum (OR = 2.20, 95% CI: 2.01–2.42, *P* < 0.001), cervical tissue blocks (OR = 2.88, 95% CI: 1.21–6.83, *P* = 0.02), and cervical secretion (OR = 2.71, 95% CI: 1.41–5.20, *P* = 0.003), especially in serum with no obvious heterogeneity.

In conclusion, our novel data demonstrate that individuals infected with *C. trachomatis* have a higher risk of cervical cancer. Therefore, it is necessary to expand *C. trachomatis* infection screening and treat women with *C. trachomatis* promptly, particularly those with human papilloma virus infections. This approach will not only protect against pelvic inflammatory disease and infertility, but may also prevent cervical cancer.

## INTRODUCTION

Cervical cancer, the 3rd most frequent cancer and the 4th leading cause of cancer death among women worldwide, accounts for nearly 10% of the total newly diagnosed cancer cases and 8% of the total cancer deaths.^1^ Human papilloma virus (HPV) is now considered the principal etiological agent in cervical cancer.^2^ However, HPV infection is mostly transient and only a small percentage of females with persistent infection eventually develop cervical cancer.^2^ Therefore, there may be other cofactors involved in enhancing the susceptibility to cervical cancer after HPV infection by facilitating HPV persistence. Behavioral and lifestyle factors and sexually transmitted infections such as bacterial vaginosis, *Chlamydia trachomatis* (*C. trachomatis*), herpes simplex virus, and human immunodeficiency virus have been identified as possible cofactors involved in cervical carcinogenesis.^3^*C. trachomatis* is one of the most common sexually transmitted pathogens in women. Epidemiological studies have shown a higher rate of *C. trachomatis* infection in patients with cervical cancer.^4^ In contrary, other researches have failed to find any association between infection with *C. trachomatis* and cervical cancer.^5–8^ For example, Tungsrithong et al^6^ in a nested case–control study in North-East Thailand indicated lack of significant effects of *C. trachomatis* infection on cervical cancer risk. Therefore, the question of whether *C. trachomatis* infection increases the risk of cervical cancer has so far not been answered and is still a matter of debate. Hence, we conducted a meta-analysis on all eligible case–control studies, cross-sectional studies, and cohort studies to systematically examine the association between *C. trachomatis* infection and cervical cancer risk. To our knowledge this is the 1st meta-analysis which provides comprehensive and quantitative evidence of the association between *C. trachomatis* infection and cervical cancer risk.

## METHODS

### Data Sources and Search Strategy

In accordance with the PRISMA guidelines, we identified published studies through a systematic review of Medline (via PubMed), Cochrane database, and EMBASE (via Ovid) from the inception to June 31, 2015, with the following search terms: (“*Chlamydia trachomatis*”) AND (“cervical carcinoma OR cervical cancer OR cancer of the cervix OR carcinoma of the cervix OR cervical neoplasm OR cervical dysplasia OR cervical intraepithelial neoplasia”). We also checked reference lists and citation histories during the search.

The following inclusion criteria were used in the meta-analysis: case–control study, cross-sectional study, or cohort study on the association between *C. trachomatis* and cervical cancers risk; the diagnosis of cervical cancer was confirmed histopathologically; sufficient sample size for estimating an odds ratio (OR) with 95% confidence intervals (CIs); studies were published in English; and the meta-analysis was restricted to original articles (no expert opinions, editorials, or reviews). Conference abstracts and other unpublished articles were also excluded. Studies were excluded if they did not meet all criteria. For multiple publications reporting the same cohort study, the largest or most recent publication was used in the meta-analysis. The approval of the study was obtained from the local research ethics committee. The protocol for this study was published on the International Prospective Register of Systematic Reviews, or PROSPERO. The registration number was CRD42014015672.

### Data Extraction and Quality Assessment

The data extraction was performed by 2 investigators independently and conflicts were resolved by consensus. For each study, the following data were extracted: first author's name, year of publication, country of origin, study year (s), study design, age range, duration of follow-up, test method for *C. trachomatis*, number of cases, and controls, as well as adjusted or crude OR with 95% CI and adjusted variables that entered into the multivariable model as potential confounders. Based on the extracted data, the quality of the included studies was evaluated by the Newcastle–Ottawa scale, a widely used tool for the quality assessment of observational or nonrandomized studies.^9^

### Statistical Analysis

The strength of association between the *C. trachomatis* and risk of cervical cancer was estimated by OR and 95% CIs. The significance of the pooled OR was determined by Z test, with *P* < 0.05 was considered significant. Heterogeneity was quantified and evaluated by the Chi-squared-based Q-test and *I*^2^ test, with *P* < 0.10 and *I*^2^ > 50% indicating evidence of heterogeneity. When the effects were assumed to be homogenous, the fixed-effects model was used (Mantel–Haenszel method).^10^ If obvious heterogeneity was present, the random-effects model was used (DerSimonian–Laird method).^11^ Subgroup analysis was conducted by histological type (squamous cell cervical carcinoma and adenocarcinoma), source of specimen (serum, cervical secretion, and paraffin-embedded tissues), and study design (univariate analysis or multivariate analysis, retrospective or prospective study). Potential publication bias was assessed by funnel plots.^12^

All analyses were conducted using Review Manager 5.3 software with forest plot, whole OR, and 95% CIs (Cochrane Review Manager Software, the Cochrane Collaboration). *P* < 0.05 was considered significant.

## RESULTS

### Literature Search Results

**FIGURE 1:**
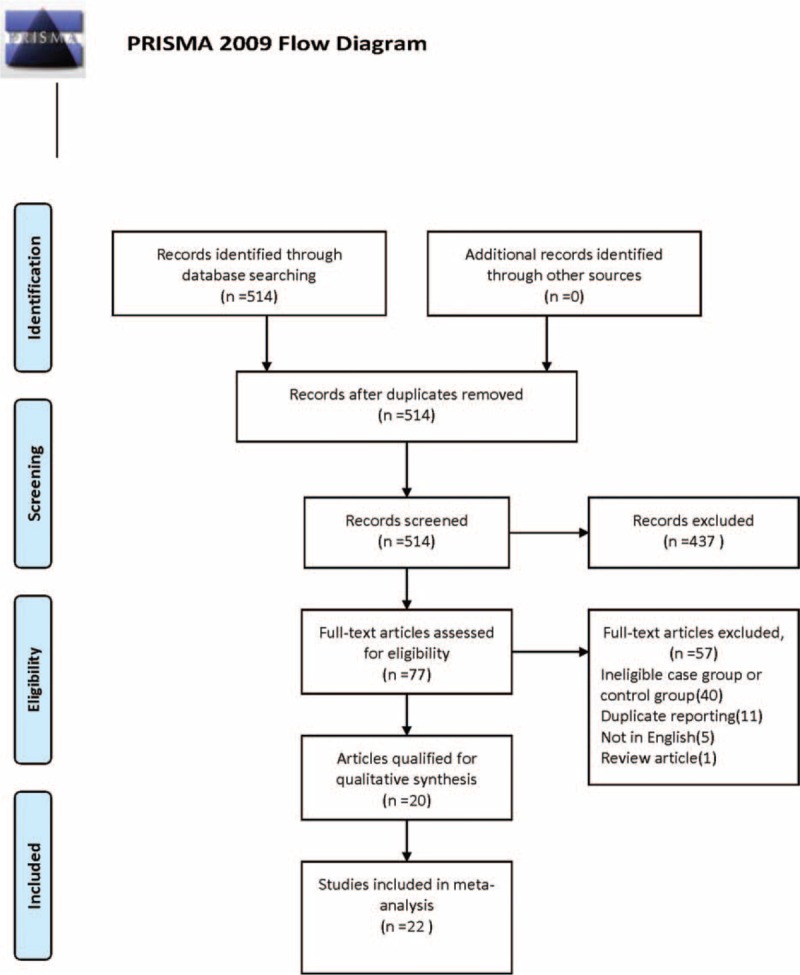
Flow chart of study selection in the meta-analysis.

The search yielded 514 records, of them, 437 were excluded as irrelevant on the basis of title and abstract. Further assessment for more detailed information identified 77 articles, of which 57 publications were excluded because of improper study design (n = 40, including 12 articles used cervical intraepithelial neoplasia as case group or control group, 3 articles used HPV-positive women as control participants), duplicate reporting (n = 11), not in English (n = 5), and review article (n = 1). Among the remaining 20 articles, 2 studies analyzed 2 distinct cohorts. One is a cross-sectional study involving 2 groups of women (group A between September 1988 and September 1993, group B between November 1995 and June 1999).^13^ The other analyzed 2 case–control studies in Spain and Colombia.^14^ The data in each of these 2 articles were extracted as 2 individual studies. Thus, 22 individual studies from 20 publications with a total of 4291 cases and 7628 controls were included in our meta-analysis. Details of the study selection process are presented in Figure 1.

### Characteristics of Included Studies

The 22 studies on *C. trachomatis* and the risk of cervical cancers were published between 1981 and 2015. Most of these studies were performed in 1 country, except 3, which involved multiple countries.^15–17^ Among those 22 studies, 19 were retrospective, while the other 3 were prospective. All studies included in this meta-analysis were prevalence studies. Most of them were case–control and cross-sectional studies, assessing *C. trachomatis* infection and cervical cancer prevalence at a given point of time. Only 3 studies had a longitudinal design and assessed prevalence data with a median followed up period of 9 years,^15^ 9.6 years,^16^ and 9 years,^8^ respectively.

All of the 22 studies were performed with univariate analysis, in which 11 of them were adopted with multivariate analysis adjusted by age, use of oral contraceptives, history of smoking, and other factors. All of the multivariate analyses were adjusted for HPV and age.

In addition, eleven studies reported the association between *C. trachomatis* and squamous cell carcinomas (SCC) and 5 studies involved adenocarcinoma in cervix.

The quality score of the included studies ranged from 6 to 9 stars according to the 9-star Newcastle–Ottawa scale. Their main characteristics are presented in the Table 1.

**TABLE 1:**
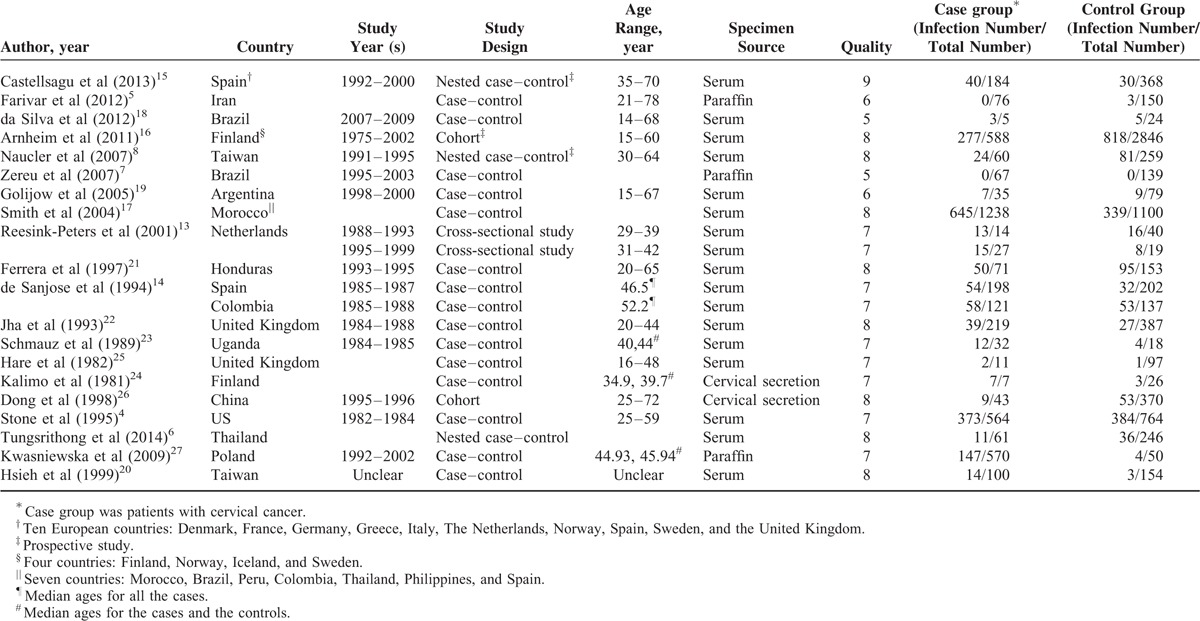
Characteristics of Included Studies on the Association Between *C. trachomatis* Infection and Cervical Cancers Risk

### Main Analysis

**FIGURE 2:**
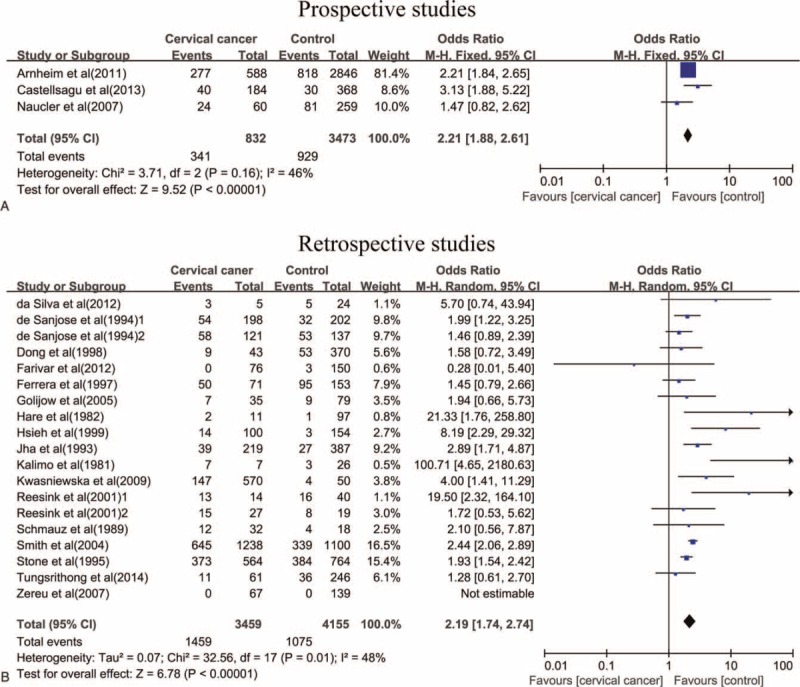
The association between *C. trachomatis* infection and the risk of cervical cancer in prospective studies and retrospective studies.

The overall prevalence of *C. trachomatis* infection in women with cervical cancer and controls in this study was 41.95% and 26.27%, respectively. Meta-analysis of total eligible studies showed that there was a significant link between *C. trachomatis* and the risk of cervical cancer in prospective studies (OR = 2.21, 95% CI: 1.88–2.61, *P* < 0.001) (Figure 2), as well as in retrospective studies (OR = 2.19, 95% CI: 1.74–2.74, *P* < 0.001) (Figure 2).

**FIGURE 3:**
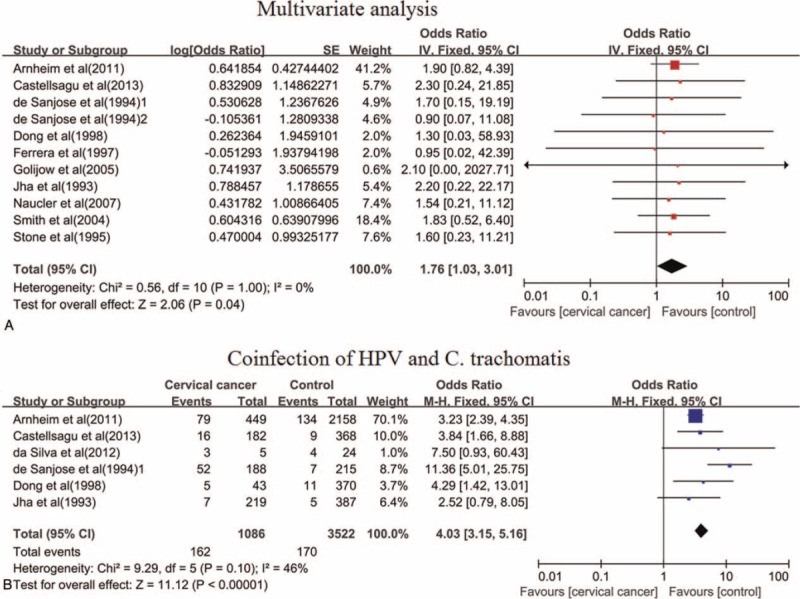
The association between *C. trachomatis* infection and the risk of cervical cancer. (A) *C. trachomatis* infection and the risk of cervical cancer by multivariate analysis. (B) Coinfection of HPV and (C) trachomatis and the risk of cervical cancer.

*C. trachomatis* was identified as an independent predictor of cervical cancer (OR = 1.76, 95% CI: 1.03–3.01, *P* = 0.04) in 11 studies using multivariate logistic regression analysis adjusted for HPV and age (Figure 3), in which 6 studies also were adjusted for oral contraceptive, 6 studies were adjusted for sexual status such as number of sexual partners, age at 1st intercourse, age at 1st birth, and number of full-term pregnancies, and 4 studies were adjusted for history of smoking. Moreover, 6 studies evaluated the coinfection of HPV and *C. trachomatis* and suggested that coinfection of HPV and *C. trachomatis* was related to a higher risk of uterine cervix cancer (OR = 4.03, 95% CI: 3.15–5.16, *P* < 0.001) (Figure 3).

### Subgroup-Analysis

**FIGURE 4:**
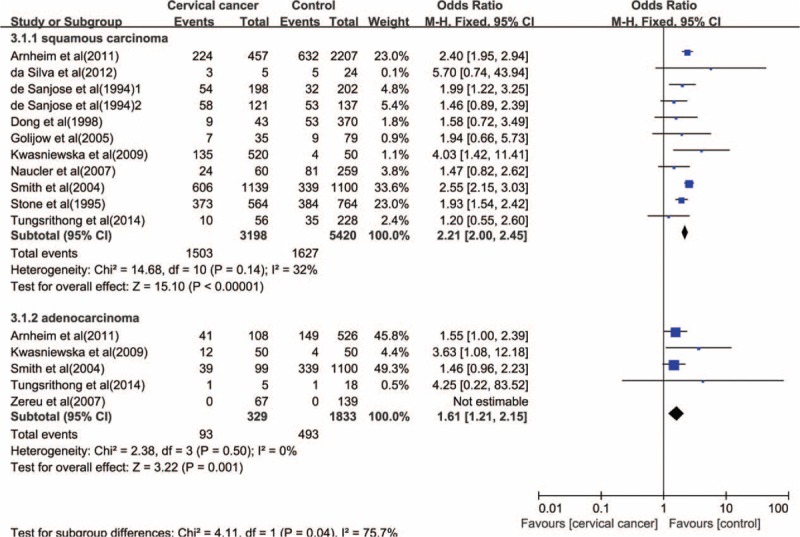
The association between *C. trachomatis* infection and the risk of cervical cancer subgrouped by histological type.

A subgroup analysis based on histological type indicated a higher risk of both SCC (OR = 2.21, 95% CI: 2.00–2.45, *P* < 0.001) and adenocarcinoma (OR = 1.61, 95% CI: 1.21–2.15, *P* = 0.001) in the cervix associated with *C. trachomatis* infection (Figure 4).

**FIGURE 5:**
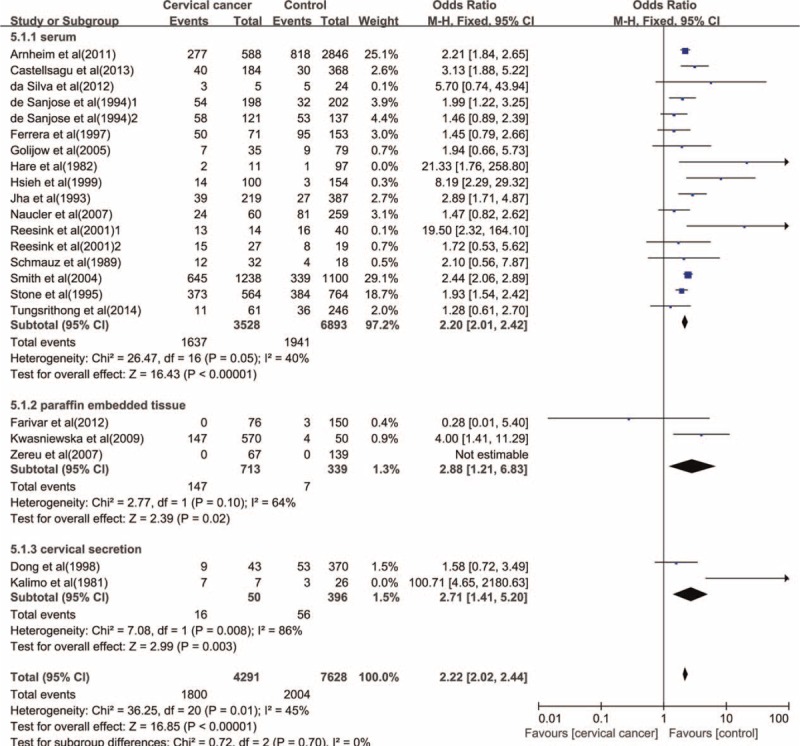
The association between *C. trachomatis* infection and the risk of cervical cancer subgrouped by specimen source.

Analysis according to specimen source indicated that an increased risk of cervical cancer was related to the infection of *C. trachomatis* detected in various specimen including serum (OR = 2.20, 95% CI: 2.01–2.42, *P* < 0.001), paraffin embedded cervical tissue (OR = 2.88, 95% CI: 1.21–6.83, *P* = 0.02), and cervical secretion (OR = 2.71, 95% CI: 1.41–5.20, *P* = 0.003) (Figure 5). However, 2 studies showed that none of cervical cancer cases was positive for *C. trachomatis* with polymerase chain reaction assay using DNA extracted from paraffin-embedded tissue specimens of patients with cervical cancer.^5,7^

### Publication Bias

**FIGURE 6:**
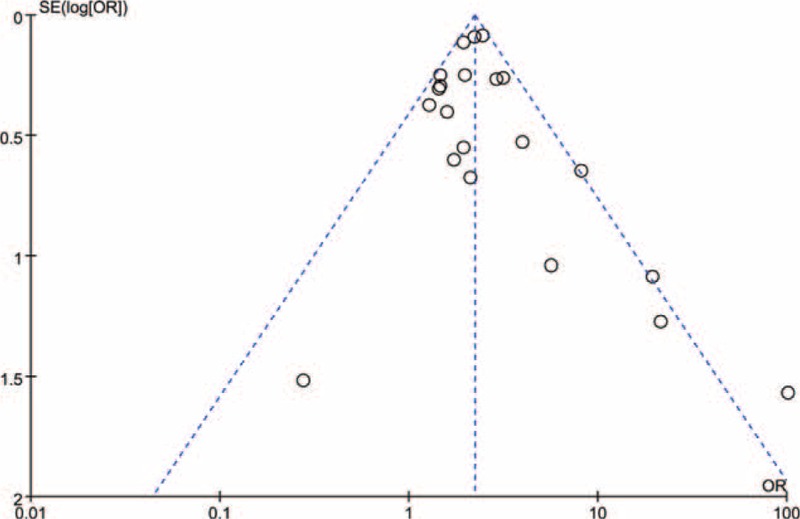
Funnel plot for the assessment of the publication bias Checklist S1 PRISMA 2009 checklist. (DOC).

Publication bias was investigated by the funnel plots. As shown in Figure 6, there was low possibility of bias on visualization of funnel plots in this meta-analysis.

## DISCUSSION

*C. trachomatis* is a gram-negative, obligate intracellular bacterium that infects human epithelial cells of the genital tract as well as ocular tissue. Genital *C. trachomatis* infection may result in pelvic inflammatory disease, ectopic pregnancy, and infertility in some cases, and is also implicated to increase the risk of cervical neoplasia.^15,16,28,29^ Whether *C. trachomatis* infection truly stimulates cervical carcinoma is one of the most pressing questions in this field of research.

In this meta-analysis of 19 retrospective studies and 3 prospective studies (with a total of 4291 cases and 7628 controls), we confirmed that *C. trachomatis* infection was significantly associated with increased risk of cervical cancer. The finding that the presence of antichlamydial heat shock protein 60–1 antibodies was associated with an increased cervical cancer risk also supports the correlation between persistent *C. trachomatis* infection and cervical neoplasia.^30^ A plausible mechanism for chlamydial infection to increase cervical cancer risk is the infection-associated inflammatory response, thus leading to reactive oxidative metabolite production, increased expression of cytokines, chemokines, and growth and angiogenic factors, decreased cell-mediated immunity, and the generation of free radicals, all of which can cause damages to DNA and impair DNA repair function resulting in genetic instability.^31,32^*C. trachomatis* infection triggered the production of supernumerary centrosomes and chromosome segregation defects, facilitated multipolar mitosis, actively promoted chromosome instability, caused multinucleation, and thereby led to transformation and tumor development.^33–36^ Additionally, *C. trachomatis* disrupted N-cadherin-dependent cell-cell junctions and caused the breakdown of the N-cadherin/β-catenin complex in primary cultures of human cervical epithelial cells and in HeLa cells.^37^ More recently, Discacciati et al^38^ found Matrix metalloproteinases-9 /Reversion-inducing Cysteine-rich protein with Kazal motifs (RECK) imbalance during cervical inflammation induced by *C. trachomatis* might play a role in cervical carcinogenesis. In the same line of evidence it was shown that infection of mice with *C. trachomatis* resulted in significantly increased cell proliferation, within the cervix, and in evidence of cervical dysplasia.^39^

Infection with HPV is established as a major cause of cervical cancer. A large body of evidence suggests that *C. trachomatis* infection may increase the risk of HPV acquisition as well as HPV persistence.^40–42^ Two recent studies have found that the history of *C. trachomatis* infection appears to increase the probability of persistent HPV infection.^43,44^ A recent prospective, population-based cohort study with up to 19 years of follow-up showed that repeated *C. trachomatis* infection increased the risk of cervical intraepithelial neoplasia 3^+^ among women with prevalent as well as persistent high-risk HPV infection.^42^ Similarly, our research indicated that coinfection of HPV and *C. trachomatis* was related to a higher risk of uterine cervical cancer, further strengthening this relationship. It may be due to 2 mechanisms. First, HPV infection in the basal keratinocytes of the mucosal epithelium requires the presence of microabrasions or altered epithelium. Chlamydial infection could possibly lead to epithelial disruption, thus, facilitating the entry of the virus.^45,46^ Second, chlamydial infection might also disturb the immune response necessary to clear the virus.^31,32^

However, the infections both of HPV and *C. trachomatis* are sexually transmitted. They have similar behavioral risk factors, such as younger age and higher numbers of sexual partners. As a result, the 2 infections could occur concurrently, rather than *C. trachomatis* infection directly affecting HPV acquisition. Epidemiologic evidence have demonstrated a higher risk of cervical cancer for women with antibodies against *C. trachomatis*, both in analyses that have adjusted for HPV infection and in stratified analyses where only HPV DNA-positive cases and controls have been included.^17,47^ In this meta-analysis, multivariate logistic regression analysis was performed in 11 studies and the meta-analysis confirmed a higher risk of developing cervical cancer in patients with *C. trachomatis* infection, even after adjustment for HPV infection. Consequently, we believe that the *C. trachomatis* infection is not likely to be a simple comorbidity with HPV infection, but should be an independent predictor for cervical cancer risk.

Results of several previous studies suggested the role of *C. trachomatis* as a carcinogenetic cofactor may be restricted to cervical SCC. Several studies found *C. trachomatis* infection was a risk factor for invasive cervical SCC, but not for adenocarcinoma.^17,48^ Similarly, 2 prospective studies showed the association between *C. pneumoniae* infection and lung SCC, but not with adenocarcinoma of the lung.^49,50^ In this study, it is an interesting result to find that *C. trachomatis* infection is associated with a higher risk in both SCC and adenocarcinoma of the cervix. Given endocervix (together with the squamocolumnar junction) is the major site of *C. trachomatis* infection in the lower genital tract, we hypothesize that *C. trachomatis* infection may play a role in the initiation of malignant transformation of the glandular endocervical epithelium via the above-outlined mechanisms related to HPV coinfection. In addition, *C. trachomatis* may play a role in the progression of adenocarcinoma by evoking the inflammatory response that damages the mucosal barrier including the basement membrane.^51^ A further study is recommended in the domain of the effect of *C. trachomatis* in adenocarcinoma carcinogenesis of cervix.

Elevated risks of cervical cancer were indicated in *C. trachomatis* infection in various specimen sources including cervical secretion, serum, and paraffin embedded cervical tissue, especially in serum with no obvious issue of heterogeneity. Therefore, *C. trachomatis* detection in serum may be a possible method to predict the risk of cervical cancer in clinic. However, 2 studies showed none of the cases was positive for *C. trachomatis* with polymerase chain reaction assay using DNA extracted from paraffin-embedded cervical tissue specimens of patients with cervical cancer. On the basis of the outcomes in this study, we believe that the polymerase chain reaction assay using paraffin-embedded cervical tissue specimens is not a reliable way to evaluate the infection of *C. trachomatis*.

Our study has some limitations. Firstly, most of our quantitative assessment studies were based on case–control studies where data on prevalence of *C. trachomatis* and cervical cancers were acquired simultaneously, rather than longitudinally. None of the studies have taken into account the association between the duration of *C. trachomatis* infection and the risk of cervical cancer. Another limitation is that a meta-analysis is impossible to tackle the problems of confounding factors that could be inherent in the included studies. Inadequate control for confounders may bias the results in overestimation or underestimation of risk estimates. Moreover, heterogeneity may be introduced because of methodological differences among studies, including different specimen sources. Although there was low possibility of bias on visualization of funnel plots in this meta-analysis, the retrieved literature might potentially not be comprehensive enough. Studies with a statistically significant effect are more likely to be published and to be cited by other authors, while the results showed no association between *C. trachomatis* and cervical cancer may be unpublished.

The underlying interaction between *C. trachomatis* and cervical cancer risk needs to be confirmed in longitudinal studies. Thus, our study calls for further investigation in more prospective studies to provide more definitive evidence concerning the role of this pathogen as a promoter of HPV-mediated cervical carcinogenesis.

In summary, this meta-analysis strengthens the evidence that infection with *C. trachomatis* could be one of the risk factor of cervical cancer. Individuals infected with *C. trachomatis* have a heightened risk of developing cervical cancer. Therefore, it is necessary to expand *C. trachomatis* infection screening and treat women with *C. trachomatis* infection timely, particularly among women at a higher risk of HPV infections. This approach will not only protect against pelvic inflammatory disease and infertility, but potentially also prevent cervical cancer and reduce the incidence of cervical cancer.

## References

[1] Jemal A Bray F Center MM Global cancer statistics. *CA Cancer J Clin* 2011; 61:69–90. 21296855 10.3322/caac.20107

[2] Schiffman M Wentzensen N Wacholder S Human papilloma virus testing in the prevention of cervical cancer. *J Natl Cancer Inst* 2011; 103:368–383. 21282563 10.1093/jnci/djq562 PMC3046952

[3] Huh WK Human papillomavirus infection: a concise review of natural history. *Obstet Gynecol* 2009; 114:139–143. 19546771 10.1097/AOG.0b013e3181ab6878

[4] Stone KM Zaidi A Rosero-Bixby L Sexual behavior, sexually transmitted diseases, and risk of cervical cancer. *Epidemiology* 1995; 6:409–414. 7548350 10.1097/00001648-199507000-00014

[5] Farivar TN Johari P Lack of association between *Chlamydia trachomatis* infection and cervical cancer – Taq Man realtime PCR assay findings. *Asian Pac J Cancer Prev* 2012; 13:3701–3704. 23098457 10.7314/apjcp.2012.13.8.3701

[6] Tungsrithong N Kasinpila C Maneenin C Lack of significant effects of *Chlamydia trachomatis* infection on cervical cancer risk in a nested case-control study in North-East Thailand. *Asian Pac J Cancer Prev* 2014; 15:1497–1500. 24606489 10.7314/apjcp.2014.15.3.1497

[7] Zereu M Zettler CG Cambruzzi E Herpes simplex virus type 2 and *Chlamydia trachomatis* in adenocarcinoma of the uterine cervix. *Gynecol Oncol* 2007; 105:172–175. 17188342 10.1016/j.ygyno.2006.11.006

[8] Naucler P Chen HC Persson K Seroprevalence of human papillomaviruses and *Chlamydia trachomatis* and cervical cancer risk: nested case-control study. *J Gen Virol* 2007; 88:814–822. 17325353 10.1099/vir.0.82503-0

[9] Wells GA Shea B O’Connell D The Newcastle–Ottawa Scale (NOS) for assessing the quality if nonrandomized studies in meta-analyses. *Dept of Epidemiology and Community Medicine, University of Ottawa: Ottawa, Canada* http://www ohri ca/programs/clinical_epidemiology/oxford.htm. [Accessed June 30, 2012]. 2011. 12.

[10] Mantel N Haenszel W Statistical aspects of the analysis of data from retrospective studies of disease. *J Natl Cancer Inst* 1959; 22:719–748. 13655060

[11] DerSimonian R Kacker R Random-effects model for meta-analysis of clinical trials: an update. *Contemp Clin Trials* 2007; 28:105–114. 16807131 10.1016/j.cct.2006.04.004

[12] Egger M Davey Smith G Schneider M Bias in meta-analysis detected by a simple, graphical test. *BMJ* 1997; 315:629–634. 9310563 10.1136/bmj.315.7109.629 PMC2127453

[13] Reesink-Peters N Ossewaarde JM Van Der Zee AG No association of anti-*Chlamydia trachomatis* antibodies and severity of cervical neoplasia. *Sex Transm Infect* 2001; 77:101–102. 11287686 10.1136/sti.77.2.101 PMC1744283

[14] de Sanjose S Munoz N Bosch FX Sexually transmitted agents and cervical neoplasia in Colombia and Spain. *Int J Cancer* 1994; 56:358–363. 8314322 10.1002/ijc.2910560311

[15] Castellsague X Pawlita M Roura E Prospective seroepidemiologic study on the role of human papillomavirus and other infections in cervical carcinogenesis: evidence from the EPIC cohort. *Int J Cancer* 2014; 135:440–452. 24338606 10.1002/ijc.28665

[16] Arnheim Dahlstrom L Andersson K Luostarinen T Prospective seroepidemiologic study of human papillomavirus and other risk factors in cervical cancer. *Cancer Epidemiol Biomarkers Prev* 2011; 20:2541–2550. 21994401 10.1158/1055-9965.EPI-11-0761

[17] Smith JS Bosetti C Munoz N *Chlamydia trachomatis* and invasive cervical cancer: a pooled analysis of the IARC multicentric case-control study. *Int J Cancer* 2004; 111:431–439. 15221973 10.1002/ijc.20257

[18] da Silva Barros NK Costa MC Alves RR Association of HPV infection and *Chlamydia trachomatis* seropositivity in cases of cervical neoplasia in Midwest Brazil. *J Med Virol* 2012; 84:1143–1150. 22585734 10.1002/jmv.23312

[19] Golijow CD Abba MC Mouron SA *Chlamydia trachomatis* and human papillomavirus infections in cervical disease in Argentine women. *Gynecol Oncol* 2005; 96:181–186. 15589598 10.1016/j.ygyno.2004.09.037

[20] Hsieh CY You SL Kao CL Reproductive and infectious risk factors for invasive cervical cancer in Taiwan. *Anticancer Res* 1999; 19:4495–4500. 10650799

[21] Ferrera A Baay MF Herbrink P A sero-epidemiological study of the relationship between sexually transmitted agents and cervical cancer in Honduras. *Int J Cancer* 1997; 73:781–785. 9399651 10.1002/(sici)1097-0215(19971210)73:6<781::aid-ijc1>3.0.co;2-z

[22] Jha PK Beral V Peto J Antibodies to human papillomavirus and to other genital infectious agents and invasive cervical cancer risk. *Lancet* 1993; 341:1116–1118. 8097804 10.1016/0140-6736(93)93128-n

[23] Schmauz R Okong P de Villiers EM Multiple infections in cases of cervical cancer from a high-incidence area in tropical Africa. *Int J Cancer* 1989; 43:805–809. 2714885 10.1002/ijc.2910430511

[24] Kalimo K Terho P Honkonen E *Chlamydia trachomatis* and herpes simplex virus IgA antibodies in cervical secretions of patients with cervical atypia. *Br J Obstet Gynaecol* 1981; 88:1130–1134. 6271164 10.1111/j.1471-0528.1981.tb01767.x

[25] Hare MJ Taylor-Robinson D Cooper P Evidence for an association between *Chlamydia trachomatis* and cervical intraepithelial neoplasia. *Br J Obstet Gynaecol* 1982; 89:489–492. 7082604 10.1111/j.1471-0528.1982.tb03643.x

[26] Dong YZ Sasagawa T Fang SY Human papillomavirus, *Chlamydia trachomatis*, and other risk factors associated with cervical cancer in china. *Int J Clin Oncol* 1998; 3:81–87.

[27] Kwasniewska A Korobowicz E Zdunek M Prevalence of *Chlamydia trachomatis* and herpes simplex virus 2 in cervical carcinoma associated with human papillomavirus detected in paraffin-sectioned samples. *Eur J Gynaecol Oncol* 2009; 30:65–70. 19317260

[28] Bebear C de Barbeyrac B Genital *Chlamydia trachomatis* infections. *Clin Microbiol Infect* 2009; 15:4–10. 19220334 10.1111/j.1469-0691.2008.02647.x

[29] Ohman H Tiitinen A Halttunen M Cytokine polymorphisms and severity of tubal damage in women with Chlamydia-associated infertility. *J Infect Dis* 2009; 199:1353–1359. 19358670 10.1086/597620

[30] Paavonen J Karunakaran KP Noguchi Y Serum antibody response to the heat shock protein 60 of *Chlamydia trachomatis* in women with developing cervical cancer. *Am J Obstet Gynecol* 2003; 189:1287–1292. 14634555 10.1067/s0002-9378(03)00755-5

[31] Simonetti AC Melo JH de Souza PR Immunological's host profile for HPV and *Chlamydia trachomatis*, a cervical cancer cofactor. *Microbes Infect* 2009; 11:435–442. 19397882 10.1016/j.micinf.2009.01.004

[32] Verteramo R Pierangeli A Mancini E Human papillomaviruses and genital co-infections in gynaecological outpatients. *BMC Infect Dis* 2009; 9:16. 19216747 10.1186/1471-2334-9-16 PMC2656516

[33] Grieshaber SS Grieshaber NA Miller N *Chlamydia trachomatis* causes centrosomal defects resulting in chromosomal segregation abnormalities. *Traffic* 2006; 7:940–949. 16882039 10.1111/j.1600-0854.2006.00439.x

[34] Johnson KA Tan M Sütterlin C Centrosome abnormalities during a *Chlamydia trachomatis* infection are caused by dysregulation of the normal duplication pathway. *Cell Microbiol* 2009; 11:1064–1073. 19290915 10.1111/j.1462-5822.2009.01307.x PMC3308718

[35] Knowlton AE Brown HM Richards TS *Chlamydia trachomatis* infection causes mitotic spindle pole defects independently from its effects on centrosome amplification. *Traffic* 2011; 12:854–866. 21477082 10.1111/j.1600-0854.2011.01204.x PMC3116664

[36] Sun HS Wilde A Harrison RE *Chlamydia trachomatis* inclusions induce asymmetric cleavage furrow formation and ingression failure in host cells. *Mol Cell Biol* 2011; 31:5011–5022. 21969606 10.1128/MCB.05734-11 PMC3233033

[37] Prozialeck WC Fay MJ Lamar PC *Chlamydia trachomatis* disrupts N-cadherin-dependent cell-cell junctions and sequesters beta-catenin in human cervical epithelial cells. *Infect Immun* 2002; 70:2605–2613. 11953402 10.1128/IAI.70.5.2605-2613.2002 PMC127927

[38] Discacciati MG Gimenes F Pennacchi PC MMP-9/RECK imbalance: a mechanism associated with high-grade cervical lesions and genital infection by human papillomavirus and *Chlamydia trachomatis*. *Cancer Epidemiol Biomarkers Prev* 2015; 24:1539–1547. 26261088 10.1158/1055-9965.EPI-15-0420

[39] Knowlton AE Fowler LJ Patel RK Chlamydia induces anchorage independence in 3T3 cells and detrimental cytological defects in an infection model. *PLoS One* 2013; 8:e54022. 23308295 10.1371/journal.pone.0054022 PMC3538680

[40] Safaeian M Quint K Schiffman M *Chlamydia trachomatis* and risk of prevalent and incident cervical premalignancy in a population-based cohort. *J Natl Cancer Inst* 2010; 102:1794–1804. 21098758 10.1093/jnci/djq436 PMC2994864

[41] Kim S Arduino JM Roberts CC Incidence and predictors of human papillomavirus-6, -11, -16, and -18 infection in young norwegian women. *Sex Transm Dis* 2011; 38:587–597. 21301390 10.1097/OLQ.0b013e31820a9324

[42] Jensen KE Thomsen LT Schmiedel S *Chlamydia trachomatis* and risk of cervical intraepithelial neoplasia grade 3 or worse in women with persistent human papillomavirus infection: a cohort study. *Sex Transm Infect* 2014; 90:550–555. 24728044 10.1136/sextrans-2013-051431

[43] Silins I Ryd W Strand A *Chlamydia trachomatis* infection and persistence of human papillomavirus. *Int J Cancer* 2005; 116:110–115. 15756673 10.1002/ijc.20970

[44] Insinga RP Perez G Wheeler CM Incidence, duration, and reappearance of type-specific cervical human papillomavirus infections in young women. *Cancer Epidemiol Biomarkers Prev* 2010; 19:1585–1594. 20530494 10.1158/1055-9965.EPI-09-1235

[45] Horvath CA Boulet GA Renoux VM Mechanisms of cell entry by human papillomaviruses: an overview. *Virol J* 2010; 7:11. 20089191 10.1186/1743-422X-7-11 PMC2823669

[46] Paba P Bonifacio D Di Bonito L Co-expression of HSV2 and *Chlamydia trachomatis* in HPV-positive cervical cancer and cervical intraepithelial neoplasia lesions is associated with aberrations in key intracellular pathways. *Intervirology* 2008; 51:230–234. 18812695 10.1159/000156481

[47] Steben M Duarte-Franco E Human papillomavirus infection: epidemiology and pathophysiology. *Gynecol Oncol* 2007; 107:S2–S5. 17938014 10.1016/j.ygyno.2007.07.067

[48] Koskela P Anttila T Bjorge T *Chlamydia trachomatis* infection as a risk factor for invasive cervical cancer. *Int J Cancer* 2000; 85:35–39. 10585579 10.1002/(sici)1097-0215(20000101)85:1<35::aid-ijc6>3.0.co;2-a

[49] Littman AJ White E Jackson LA *Chlamydia pneumoniae* infection and risk of lung cancer. *Cancer Epidemiol Biomarkers Prev* 2004; 13:1624–1630. 15466979

[50] Laurila AL Anttila T Laara E Serological evidence of an association between *Chlamydia pneumoniae* infection and lung cancer. *Int J Cancer* 1997; 74:31–34. 9036866 10.1002/(sici)1097-0215(19970220)74:1<31::aid-ijc6>3.0.co;2-1

[51] Quint KD de Koning MN Geraets DT Comprehensive analysis of human papillomavirus and *Chlamydia trachomatis* in in-situ and invasive cervical adenocarcinoma. *Gynecol Oncol* 2009; 114:390–394. 19500822 10.1016/j.ygyno.2009.05.013

